# Tumor Suppressors—HTRA Proteases and Interleukin-12—in Pediatric Asthma and Allergic Rhinitis Patients

**DOI:** 10.3390/medicina56060298

**Published:** 2020-06-17

**Authors:** Joanna Renke, Eliza Wasilewska, Sabina Kędzierska-Mieszkowska, Katarzyna Zorena, Sylwia Barańska, Tomasz Wenta, Anna Liberek, Danuta Siluk, Dorota Żurawa-Janicka, Aleksandra Szczepankiewicz, Marcin Renke, Barbara Lipińska

**Affiliations:** 1 Department of General and Medical Biochemistry, University of Gdańsk, Wita Stwosza 59 80-308 Gdańsk, Poland; sabina.kedzierska-mieszkowska@ug.edu.pl (S.K.-M.); tomasz.wenta@ug.edu.pl (T.W.); dorota.zurawa-janicka@ug.edu.pl (D.Ż.-J.); barbara.lipinska@ug.edu.pl (B.L.); 2 Department of Allergology, Medical University of Gdańsk, Dębinki 7, 80-210 Gdańsk, Poland; ewasilewska@gumed.edu.pl; 3 Department of Immunobiology and Environmental Microbiology Medical University of Gdańsk, Dębinki 7, 80-210 Gdańsk, Poland; kzorena@gumed.edu.pl; 4 Department of Bacterial Molecular Genetics University of Gdańsk Wita Stwosza 59, 80-308 Gdańsk, Poland; sylwia.baranska@ug.edu.pl; 5 Faculty of Health Sciences with Subfaculty of Nursing, Medical University of Gdańsk, Tuwima 15, 80-210 Gdańsk, Poland; alib@gumed.edu.pl; 6 Department of Biopharmaceutics and Pharmacodynamics, Medical University of Gdańsk, Hallera 107, 80-416 Gdańsk, Poland; danuta.siluk@gumed.edu.pl; 7 Laboratory of Molecular and Cell Biology, Department of Pediatric Pulmonology, Allergy and Clinical Immunology, Poznan University of Medical Sciences, 60-512 Poznan, Poland; alszczep@ump.edu.pl; 8 Department of Occupational, Metabolic and Internal Diseases, Medical University of Gdańsk, Powstania Styczniowego 9B, 81-519 Gdynia, Poland; mrenke@gumed.edu.pl

**Keywords:** HTRA proteases, Il-12, allergy, oncogenesis, mast cells

## Abstract

*Background and objective*: Allergy belongs to a group of mast cell-related disorders and is one of the most common diseases of childhood. It was shown that asthma and allergic rhinitis diminish the risk of various cancers, including colon cancer and acute lymphoblastic leukemia. On the other hand, asthma augments the risk of lung cancer and an increased risk of breast cancer in patients with allergy has been observed. Thus, the relation between allergy and cancer is not straightforward and furthermore, its biological mechanism is unknown. The HTRA (high temperature requirement A) proteases promote apoptosis, may function as tumor suppressors and HTRA1 is known to be released by mast cells. Interleukin-12 (Il-12) is an important cytokine that induces antitumor immune responses and is produced mainly by dendritic cells that co-localize with mast cells in superficial organs. *Material and methods*: In the present study we have assessed with ELISA plasma levels of the HTRA proteins, Il-12, and of the anti-HTRA autoantibodies in children with allergy (40) and in age matched controls (39). Children are a special population, since they usually do not have comorbidities and take not many drugs the processes we want to observe are not influenced by many other factors. *Results*: We have found a significant increase of HTRA1, 2 and 3, and of the Il-12 levels in the children with atopy (asthma and allergic rhinitis) compared to controls. *Conclusion*: Our results suggest that the HTRA1–3 and Il-12 levels might be useful in analyzing the pro- and antioncogenic potential in young atopic patients.

## 1. Introduction

The influence of allergic reactions on carcinogenesis has become an important scientific issue. The link between allergies, tolerance and cancer risk is deeply analyzed to find some new solutions in preventing or even treating neoplastic diseases [1,2,3,4]. There are four main allergy types according to Gell–Coombs classification. Among them the IgE-dependent reactions are the best understood also from the oncological point of view [1,2].

IgE-mediated allergy manifesting as atopic asthma (AA) or allergic rhinitis (AR) is one of the most common diseases of childhood. Children are a unique population and a disease model very interesting to analyze since they usually do not have comorbidities and take not many drugs, the processes one wants to observe are not influenced by so many unpredictable other factors, as it happens in adult patients’ analysis. Asthma is a heterogenous clinical syndrome of airway obstruction, hyperresponsiveness and inflammation involving mast cells, with various responsiveness to therapeutic agents [1]. AR is a consequence of immediate hypersensitivity reactions to the inhaled allergens, connected with mucosal edema, mucus secretion and stuffy nose [2]. Both allergies belong to a group of mast cell-related diseases mediated by IgE and are connected with a not well described cytokine imbalance which over the long-term might influence the processes of oncogenesis. Associations between allergies and cancer have been analyzed in recent reviews of the relevant quantitative studies [3,4]. Cui and Hill (2016) reported that in most studies an inverse association between atopy and non-Hodgkin lymphoma or colorectal cancer was found [3]. A protective influence of allergy of any type against glioma and adult acute lymphoblastic leukemia was also observed. Protective effects of atopic diseases against pancreatic cancer have been found in a group of case-control studies. On the other hand, asthma appeared to be a risk factor for lung cancer [3]. A positive association between asthma and lung cancer was also found in a group of studies analyzed by Muir et al. [4], however, such association was not observed in another data set [4]. Many studies also indicated the increased risk of breast cancer in patients with AR and atopic dermatitis [5]. It should be also noted that in an animal model high innate IgE levels prolonged survival of mice with mammary tumors [6]. An inverse association between ductal adenocarcinoma of the pancreas and asthma and nasal allergies has been described by Gomez-Rubio et al. [7]. A retrospective allergy study of patients diagnosed with lung, colon, breast or skin cancer indicated that the overall incidence of allergies, particularly AR, was lower in patients with some types of cancer [8].

The nature of biological relationships between cancers and allergies is not well understood [9]. The most obvious is a link between chronic inflammation accompanying allergic diseases and cancer since it is known that persistent inflammation promotes tumorigenesis [10]. However, the observed negative relationship between allergy and a majority of studied cancers seems to stay in contradiction to the aforementioned association. It has been proposed that the decreased cancer risk associated with allergic diseases could be due to increased immunosurveillance and activation of cellular defense mechanisms, including apoptosis [9,11]. Another aspect is that allergy is inevitably connected with mast cells. In various types of cancer infiltration of mast cells (MCs) is correlated with worse prognosis [12]. MCs, strongly influenced by antimediator treatments, are in the center of immunological reactions and their activation may reflect the arousal of other immune cells responsible for triggering the pro- or antioncogenic processes [13].

To gain insight into possible biological mechanisms underlying the associations between allergy and cancer, we studied serum levels of the high temperature requirement A (HTRA) proteases and interleukin-12 (Il-12) in children diagnosed with allergy. We focused on the HTRA proteases and Il-12 since they are known to function as tumor suppressors [14,15].

There are four human members of the HTRA family, HTRA1–4. In this study we focused on three of them due to technical reasons—the HTRA4 was newly described.

The majority of HTRA1 pool is secreted into extracellular space while the remaining fraction localizes to the cytoplasm [14,16,17]. It has been demonstrated that HTRA1 is involved in induction of cell death by apoptosis via degradation of X-linked inhibitor of apoptosis protein (XIAP) and by anoikis via modulation of the epidermal growth factor receptor (EGFR) [14]. Interestingly, the human MCs release HTRA1 constitutively, independently of degranulation [16].

HTRA2 is a mitochondrial protein which upon stress may be released to cytosol and trigger apoptosis of damaged cells via interaction with the inhibitor of apoptosis proteins (IAP), including XIAP [14]. In response to cell detachment HTRA2 is released from mitochondria to cytosol and promotes anoikis [18]. It participates in metabolism of amyloid precursors and its dysfunction leads to neurodegenerative diseases, like Parkinson’s and Alzheimer’s [19,20].

HTRA3 may exist in a cell as a long or short isoform, but the physiological meaning of this structural difference is unknown [14]. It is a proapoptotic protein and, similarly to HTRA1, an inhibitor of the TGF-β signaling pathway [21]. Downregulation of HTRA3 was noted in ovarian tumors, endometrial and lung cancers [22], and antagonism of HTRA3 and TGFβ1 (Tumor growth factor Beta) contributes to metastasis of non-small cell lung cancer [23].

Interleukin-12 is well recognized as an agent preventing cancer initiation, growth and metastasis [15]. This cytokine is produced by macrophages, MCs and dendritic cells that colocalize with MCs in superficial organs. It promotes proliferation, activation and cytotoxicity of T and NK cells, stimulates production and activation of Th1, stimulates the production of some IgG antibodies and blocks IgE production. Il-12 has a strong antiangiogenic potential through stimulation of IFN-γ (interferon gamma) and TNF-α (Tumor necrosis factor alpha) release, thus is proposed to be used in oncologic treatment [15]. Il-12 is also used as a marker of omalizumab (anti-IgE monoclonal antibody) treatment in severe asthmatics. Responders showed significantly higher baseline serum levels of Il-12 compared to non-responders [24] and its level decreased during treatment.

The aim of the present study was to analyze serum levels of tumor suppressors—HTRA1, HTRA2, HTRA3 proteins and Il-12—in pediatric patients and control group so as to consider its role in assessing the antioncogenic potential in atopic asthma (AA) and allergic rhinitis (AR).

## 2. Materials and Methods

### 2.1. Study Design

In this prospective case-control study, 79 children were included. The study was performed in compliance with the Code of Ethics of the World Medical Association (Declaration of Helsinki). The project was approved by the Bioethical Committee of the Medical University of Gdańsk (No. NKBBN 520-215/2018).

### 2.2. Patients

Forty allergic patients of Caucasian origin ranging from 6 to 18 years of age were recruited to the study from Outpatient Department of Allergology, Medical University of Gdansk. Asthma diagnosis was made according to GINA (Global Initiative Against Asthma) recommendation, and AR according to ARIA (Allergic Rhinitis and its Impact on Asthma) [25,26]. Lung function test was performed using a Pneumo Screen (Jaeger, Germany) spirometer according to ERS/ATS guidelines [27]. The highest value of forced expiratory volume in 1 second (FEV1) and forced vital capacity (FVC) expressed as percent predicted value (%pv) from correct acceptable attempts were included into the analysis. Atopic background was confirmed with positive skin prick test result to at least one aero-allergen (Dermatophagoides pteronyssinus, Dermatophagoides farinae, cat, dog, Alternaria alternata, Cladosporium herbarum, pollen: grass mix, rye, birch pollen, alder, hazel—Allergopharma, Reinbek, Germany).

### 2.3. Control Group

Control group consisted of 39 healthy subjects of Caucasian origin ranging from 1 to 18 years of age. Control subjects without current and past asthma and AR symptoms were recruited from the same geographic region as diagnosed patients.

### 2.4. HTRA and IL-12 Levels Analysis

The HTRA proteins and anti-HTRA IgGs were detected using enzyme linked immunosorbent assay (ELISA) and confirmed by western blotting. ELISA with high-binding plates coated with appropriate recombinant HTRA protein (a capture antigen) and rabbit polyclonal antibodies to the human HTRA1-3 proteins were used. To visualize binding of the human or rabbit anti-HTRA antibodies to a recombinant HTRA protein, SDS-PAGE (sodium dodecyl sulfate-polyacrylamide gel) electrophoresis of the protein was performed according to Laemmli et al. [28]. Subsequently, western blotting was performed as described by Harlow et al. [29], using the anti-human IgG or anti-rabbit IgG antibodies. 

Serum Il-12 levels were measured by immunoenzymatic ELISA method (Quantikine High Sensitivity Human by R&D Systems, Minneapolis, MN, USA) according to manufacturer protocol. Minimum detectable concentrations were determined by the manufacturer as 1.0 pg/ml. Intra-assay and inter-assay coefficient of variation (CV) was 1.2% and 7.1%, respectively. Precision performances of the assays were determined on 20 replicates from the quality control data of the laboratory. Absorbance was read at 450 nm on an automated plate reader (ChroMate 4300, Awareness Technology, Inc., Palm City, FL, USA). The reference curve was prepared according to the manufacturer’s recommendations.

### 2.5. Statistical Analysis

The data concerning the Il-12 levels were analyzed using the Mann–Whitney U test. The data concerning the HTRA1–3 levels were analyzed with the use of the unpaired Student’s *t*-test. Normality of the distribution of the data was checked with the D’Agostino–Pearson test and equality of the variances with the *F*-test. If variances were found significantly different then the unpaired *t*-test with Welch’s correction was performed. In the case of non-Gaussian distribution for group comparisons U Mann-Whitney test was used. Probability (*p*) values *p* < 0.05 were considered statistically significant.

## 3. Results

### 3.1. Patients Characteristics

The general characteristics of patients enrolled in the study are presented in Table 1. Student’s *t*-test analysis of differences in spirometry parameters)

### 3.2. Serum HTRA Analysis

**Figure 1:**
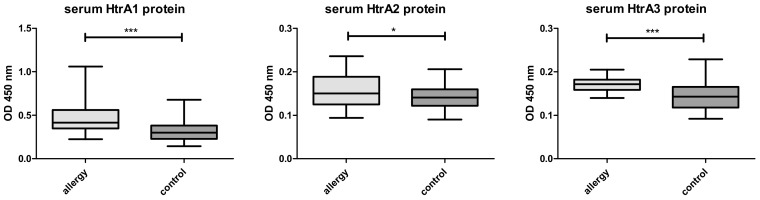
HTRA 1, 2 and 3 levels in children with allergy (n = 40) and in control group (n = 39). All serum samples were analyzed in duplicate. *** denotes *p* < 0.001, * denotes *p* = 0.021.

**Figure 2:**
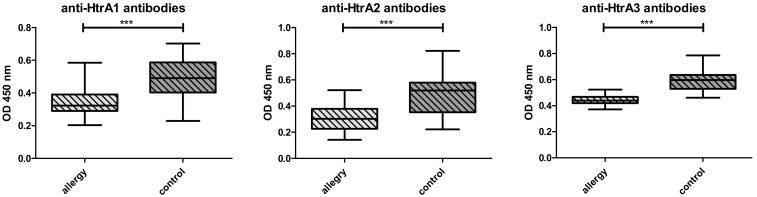
Comparison of the serum antibody levels against the HTRA proteins in children with allergy (n = 40) and in controls (n = 39), assayed by ELISA. All serum samples were analyzed in duplicate. *** denotes *p* < 0.001.

**Figure 3:**
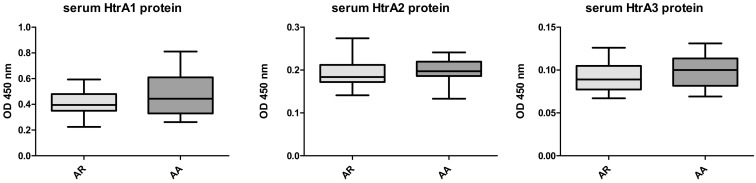
HTRA 1–3 levels in patients with allergic rhinitis (AR) and atopic asthma (AA)—nonsignificant.

**Figure 4:**
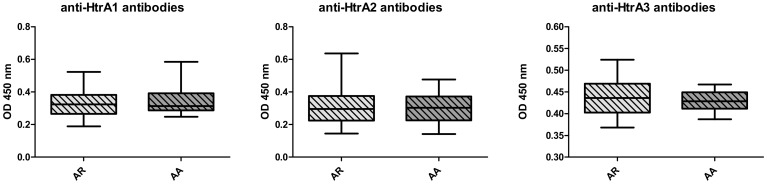
The serum antibody levels against the HTRA proteins in allergic rhinitis (AR) vs atopic asthma (AA) groups—nonsignificant.

In allergic patients the levels of HTRA1, 2 and 3 proteins were significantly higher (Figure 1) than in the control group. The autoantibodies against HTRA1–3 were lower in allergics than in the controls (Figure 2). No differences concerning both the protein levels or autoantibodies between the groups with AA vs AR were noted (Figure 3 and Figure 4).

### 3.3. Serum Il-12 Analysis

**Figure 5:**
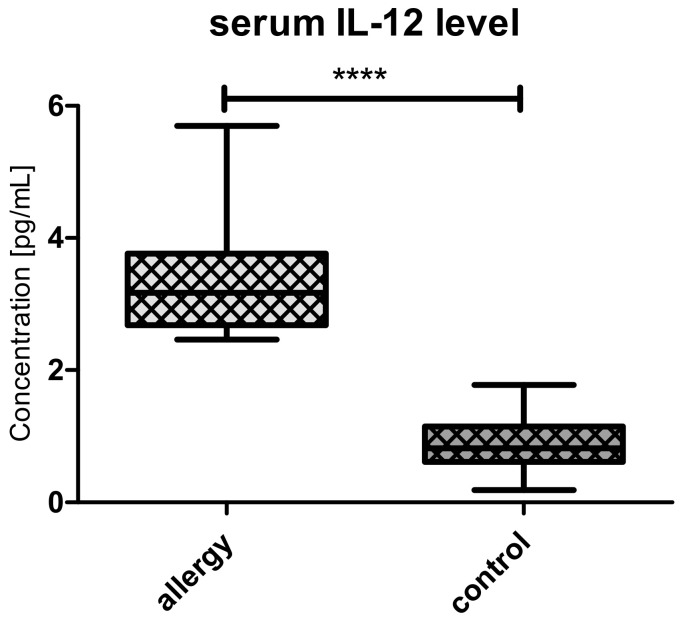
The levels of Il-12 in allergy (n = 21) and controls (n = 39). **** denotes *p* < 0.0001

Il-12 levels were significantly higher in allergic group than in controls (Figure 5).

## 4. Discussion

The main finding of this study is that the levels of tumor suppressors, the apoptotic regulators of HTRA family and of interleukin 12 known to inhibit tumorigenesis and induce regression of established tumors, were importantly higher in children with IgE-dependent allergy. It correlates with the general clinical observation that atopic patients are less prone to develop certain types of neoplasms [4]. These results were found interesting since the HTRA protein levels and anti-HTRA antibodies in mast cell–related disorders were analyzed in our earlier study concerning pediatric patients with cutaneous mastocytosis (CM), a disease due to abnormal accumulation of MCs [30]. This lymphoproliferative mast cell disorder in adulthood is always linked with augmented risk of hematologic malignancies [31]. This fact can be easily proved by molecular biology findings. The important role of MCs in angiogenesis due to release of VEGF, platelet growth factor, Il-6, Il-8 and proteases contributes to oncogenesis. Other factors released from MC, e.g., histamine and heparin, may also promote the adhesion between tumor and endothelial cells [32,33]. MCs support tumor invasiveness by releasing a broad range of matrix metalloproteinases (MMPs) [13]. Our data concerning the serum level of HTRA family apoptosis regulators in pediatric CM, among them HTRA1 constitutively released from MCs, has shown similar levels of HTRA1, 3 and 4 proteins as in healthy, pediatric group [30]. Unexpectedly, it was only HTRA2 serum level that was higher in CM than in controls. The mechanism of HTRA2 release from any cell into extracellular space is unclear. Nevertheless, while in CM as in a lymphoproliferative disorder, the release of HTRA2 from abnormal MCs into extracellular space (ECS) might be expected; the reason why HTRA2 is assessed in serum in higher amounts than in controls in allergy remains unclear. Moreover, in a previous study we have found that anti-HTRA1, 3 and 4 antibody levels were importantly higher in CM group than in controls in accordance with its recently proved presence in ECS [19,30]. Anti-HTRA2 levels were at the same range as in controls [27]. The absence of augmentation in HTRA protein levels and strong cell death inductors in CM patients might be consistent with the role of MCs in tumor promotion and observation of higher incidence of solid tumors in mastocytosis patients [34].

The correlation between MCs activity and Il-12 level was not analyzed in available literature, however it is closely correlated with NK and T-cell cytotoxicity, tumor necrosis factor (TNFα) and interferon gamma (IFNγ) activities known to play crucial roles in tumor microenvironment with MCs derived cytokines [14,35]. Antioncogenic properties of Il-12 are appreciated and used as a therapeutic tool [15].

The clinical signs of IgE-related allergy are inevitably due to activation of MCs through FcεRI (high-affinity IgE receptor) (and the release of mediators responsible for many immunological reactions, inflammation and vascular permeability [14]. This may influence some oncogenesis processes in the course of chronic inflammation.

Taking under account the previous study of CM, we were interested to find out how the augmented activation of MC by FcεR influences HTRAs and one of the most promising antitumor interleukins—Il-12. Our new results in allergic patients may suggest that HTRA proteins and Il-12 may contribute to the lower incidence of cancers in patients with IgE-mediated allergic diseases observed by scientists [8].

The reason for such results might be laid in regulation of TGFβ, in which HTRA and Il-12-related cytokines—TNF α and IFN γ—are involved [36]. We suggest that the higher level of HTRA3 and HTRA1 in children with atopy and asthma might be connected with their known interactions with TGFβ. TGFβ regulates multiple cellular processes, among them fibroblast activation and extracellular matrix organization, and it is necessary for lung branching and alveolarization. TGFβ is crucial in asthmatic inflammation and pivotal to the development of tumor promoting microenvironment in lung cancer tissue [36]. A metanalysis suggests that the TGFβ C509T polymorphism may predispose to asthma [37]. A recent big study of Liu et al. 2018 has shown as well that TGFβ1 polymorphism might be a risk factor for asthma, especially in children [38]. As it was shown, TGFβ expression is increased in bronchial specimens of asthmatic patients and plays multifunctional roles in T-cell differentiation, and the higher levels of its inhibitors, among them HTRA1 and HTRA3 proteases, might also be expected.

Higher levels of HTRA1–3 proteins than controls in allergy and not in mastocytosis may suggest that synthesis of these proteins is more intensive in allergic disorders. The study demonstrated that HTRA1-3 levels were not dependent on the number of MCs (higher in CM) but more likely on its activity. Higher levels of anti-HTRA antibodies in CM and lower in allergy may reflect differences in MCs burden or the time of exposure to interactions with immunologic cells while present in ECS.

The levels of different cytokines were widely analyzed in asthma, even in pediatric populations. The study of Cui et al. [1] has shown that Il-12 levels were elevated after treatment of children with moderate-to-severe asthma, thus suggesting that it is associated with cellular immune function and lung function [1]. It is widely accepted that cytokine imbalance plays an important role in asthma pathogenesis, less sure is whether it plays a role in carcinogenesis. Our results confirmed the elevated levels of Il-12 in pediatric patients with asthma and allergic rhinitis. This phenomenon needs further study and long-term observation of this group of patients.

## 5. Conclusions

Our study is a preliminary approach to the topic of assessing the risk of malignancy in atopic patients on the basis of apoptotic regulators and antitumor cytokines. In our opinion both Il-12 levels and serine proteases of HTRA family might be applied to this task.

## Figures and Tables

**Table 1:** General characteristics of the analyzed population.

	Patients with Allergy (n = 40)	Control Group (n = 39)
Age (years)		
range (median)	6–18 (13.5)	1–17 (9)
Gender F/M (n)	18/22	21/18
Diagnosis		
Atopic Asthma (n)	19	-
Allergic Rhinitis (n)	21	-
Positive Skin Prick Test (n)	40	-
Spirometry pv (mean ± SD)		
FEV1% (*p* < 0.001; T = − 7,5; df = 77)	83.1 ± 13.8	101.7 ± 7.1
FVC% (*p* < 0.001; T = − 3,5; df = 77)	90.1 ± 13.4	100.1 ± 11.9
FEV1/FVC (*p* < 0.001; T = − 6,41; df = 77)	87.7 ± 10.5	101.3 ± 8.2

*p*—probability value, SD—Standard Deviation, T-distribution, df—degrees of freedom.
